# Bilingualism: A Neurocognitive Exercise in Managing Uncertainty

**DOI:** 10.1162/nol_a_00044

**Published:** 2021-11-11

**Authors:** Jason W. Gullifer, Debra Titone

**Affiliations:** Department of Psychology, McGill University, Centre for Research on Brain, Language and Music, Montréal, Canada

**Keywords:** bilingualism, neurocognition, adaptation, uncertainty, entropy, individual differences

## Abstract

Bilinguals have distinct linguistic experiences relative to monolinguals, stemming from interactions with the environment and the individuals therein. Theories of language control hypothesize that these experiences play a role in adapting the neurocognitive systems responsible for control. Here we posit a potential mechanism for these adaptations, namely that bilinguals face additional language-related uncertainties on top of other ambiguities that regularly occur in language, such as lexical and syntactic competition. When faced with uncertainty in the environment, people adapt internal representations to lessen these uncertainties, which can aid in executive control and decision-making. We overview a cognitive framework on uncertainty, which we extend to language and bilingualism. We then review two “case studies,” assessing language-related uncertainty for bilingual contexts using language entropy and network scientific approaches. Overall, we find that there is substantial individual variability in the extent to which people experience language-related uncertainties in their environments, but also regularity across some contexts. This information, in turn, predicts cognitive adaptations associated with language fluency and engagement in proactive cognitive control strategies. These findings suggest that bilinguals adapt to the cumulative language-related uncertainties in the environment. We conclude by suggesting avenues for future research and links with other research domains. Ultimately, a focus on uncertainty will help bridge traditionally separate scientific domains, such as language processing, bilingualism, and decision-making.

## INTRODUCTION

Bilinguals, people who know and use more than one language, have different linguistic experiences relative to monolinguals, who know only one language. These experiences stem from different interactions with their environments and the individuals therein. Whether someone is trying to decipher multilingual signs at high speeds on the highway, order coffee in a bilingual city, or communicate academic research to multilingual peers, the people involved in these interactions bring to the table their individual levels of language knowledge, language fluency, language preferences, overt goals, and covert intentions. Bilingual environments thus have fluctuating language demands (Anderson et al., 2018; Beatty-Martinez et al., 2020; Bice & Kroll, 2019; Grosjean, 2001; Gullifer et al., 2021; Gullifer & Titone, 2020a; López, 2020; López et al., 2020; Tiv et al., 2020b), which correspond to a set of cognitive, linguistic, and social uncertainties. Individuals must resolve or adapt to these uncertainties by tuning the neurocognitive systems responsible for language and cognitive control (Abutalebi & Green, 2016; Green & Abutalebi, 2013; Green & Wei, 2014).

Fundamentally, bilinguals make choices about which languages to speak when and with whom, and they must appropriately engage their language systems to realize these choices. Even after an intended language has been chosen, bilinguals continue to experience lasting cross-language activation and competition within their linguistic subsystems that can help or hinder comprehension and production (Gullifer et al., 2013; Gullifer & Titone, 2019). To produce a word or utterance in the intended language, bilinguals must resolve this competition, otherwise bilingual speech would exhibit rampant language errors. However, bilinguals rarely commit this type of speech error (Poulisse, 2000); they have no apparent issue producing the intended language. At the same time, there is evidence that some types of cross-language competition may never be fully resolved, even in language production (Jacobs et al., 2016).

One thought is that bilinguals recruit a form of cognitive control to help manage cross-language competition. *Cognitive control* is an umbrella term that refers to a set of latent cognitive functions that may be differentially recruited by cognitive tasks (e.g., inhibition, monitoring, updating, planning, switching; Miyake et al., 2000). Thus, the psychological mechanisms implicated in bilingual cognitive control are many and are frequently under debate (Costa et al., 2008; Declerck, 2020; Declerck et al., 2019; Gullifer & Titone, 2020b; Kang et al., 2020; Ma et al., 2016). Neurally, there appears to be a broad network of brain regions involved in language control, including cortical regions (notably, frontal cortex), subcortical regions (notably, dorsal striatal regions: caudate and putamen), and cerebellar regions. Regular recruitment of these systems over the lifespan leads to adaptive changes in behavior and underlying brain architecture, including gray and white matter structures (Abutalebi & Green, 2016; Bialystok, 2017; Pliatsikas, 2020).

However, there are several mutually nonexclusive points of debate surrounding these issues (Baum & Titone, 2014; de Bruin & Della Sala, 2019; Hilchey & Klein, 2011; Leivada et al., 2020). Stable patterns of adaptations are not consistently observed across studies and geographic locations. This variation has led to questions about whether the observed cognitive adaptations are due to low-powered investigations, questionable research practices, and human biases (de Bruin et al., 2015; Donnelly et al., 2019; Lehtonen et al., 2018; Paap et al., 2015, 2019) or whether they are small effects that vary with respect to the population involved (Bialystok et al., 2016; Gullifer & Titone, 2020b). While some critiques of methodological practices are valid, in our view, they cannot simply explain away an entire body of evidence, particularly when emerging studies with extremely high bars for methodological rigor largely confirm prior results (Gullifer, Pivneva, et al., 2021; Gullifer & Titone, 2020b).

Of greater relevance, there are several substantive questions that warrant further investigation. Which neurocognitive mechanisms are involved in these adaptations, and are they specific to language (Declerck, 2020; Gullifer & Titone, 2020b; Paap et al., 2019; Pivneva et al., 2014; Takahesu Tabori et al., 2018)? How do these adaptations change over time, during language learning/acquisition (Bogulski et al., 2019; Byers-Heinlein et al., 2017; Chai et al., 2016), and as a function of learning, usage, and immersion (DeLuca et al., 2019; Pliatsikas, 2020)? Finally, there are questions about which bilingual experiences are important, and how the context of language usage, which might differ according to geographical locations, impacts these adaptations (Adler et al., 2020; Beatty-Martínez & Dussias, 2017; Gullifer, Kousaie, et al., 2021; López et al., 2020; Zirnstein et al., 2019).

### The Current Study

In this review, we propose that centralizing bilingualism within a cognitive-linguistic framework that emphasizes the more general idea of *uncertainty* provides a fruitful way to think about these issues. Uncertainty is a key principle in many domains of science, and it figures centrally in neurobiology, attention, decision-making, and language processing. In the past, the systems and principles underlying language were often studied separately from those underlying cognition. However, the human neurocognitive system is best viewed as a set of interactive and adaptive systems, and bilingualism has likely played a central role in elucidating the linkages between language and other cognitive systems (Kroll et al., 2014). Namely, the cognitive neuroscience of bilingualism is beginning to reveal the ways in which the cognitive systems adapt to cope with the demands of the environment, which will differ according to several factors and across geographical locations. Here, we first describe a cognitive-linguistic perspective on uncertainty, in which uncertainty becomes a facet between these two fields. We then highlight the advantages of this approach, that is, how each field can mutually benefit the other, and describe some recent applications of uncertainty to the study of bilingualism. Lastly, we pose some directions for future research.

## A COGNITIVE-LINGUISTIC PERSPECTIVE ON UNCERTAINTY

As humans, we encounter various forms of uncertainty as we move through our daily lives. These types of uncertainty occur with varying frequency (some occurring every day, others once in a lifetime). They also carry consequences of varying magnitudes: What will I cook for dinner; can I afford to cook dinner? Should I speak in English or French to this new person? When will a vaccine be universally available to curb a global pandemic? Some uncertainties may be unexpected, such as the onset of the COVID-19 global pandemic. Other uncertainties may be expected, for example, in the case that money is routinely tight at the end of the month, or the possibility of using either language that you know within a highly bilingual environment. Individuals must adapt their decision-making processes and underlying neurocognitive mechanisms to cope with such uncertainties. Language provides an optimal domain in which to study the impact of uncertainty because linguistic environments are rife with uncertainties at multiple levels of representation. Crucially, people who are bilingual experience all the typical uncertainties associated with language, as well as the added uncertainty of choosing a particular language according to the demands of particular moments.

Uncertainty can be measured with a quantity known as *entropy*, a concept from physics and information theory. Physically, entropy is a property of systems that is proportional to the log-number of different configurations, or states, of those systems. Claude Shannon, a founder of information theory, adapted entropy as a means to quantify the uncertainty of signals as proportional to the number of potential signals that could have been received (Shannon, 1948; for a succinct history of entropy, see Hirsh et al., 2012). This uncertainty, in turn, relates to the potential of a signal to carry information (surprisal). If a particular signal (or event) is highly likely, it is not very surprising and carries little information. In contrast, an unlikely event is more surprising and carries more information.

### Uncertainty at the General Cognitive Level

Uncertainty and entropy have been used in psychological and neurocognitive theories such as the psychological entropy framework (Hirsh et al., 2012) and the free energy principle (Feldman & Friston, 2010; Friston, 2010; Peters et al., 2017), in the domains of decision-making, stress, and anxiety. Fundamentally, these perspectives state that self-organizing complex systems, like the brains or minds of humans, must maintain equilibrium within an ever-shifting environment. They do so by limiting the possible set of internal states that can be occupied by these systems (e.g., sensory states, brain states), which helps to minimize surprisal for events that occur in the external environment. Failures to adapt to the environment may lead to stress and anxiety, and, over the long term, other diseases (Peters et al., 2017).

People are sensitive to the statistical regularities that occur in their environments, and they build expectations or heuristics that allow them to make inferences about upcoming information or rewards. In contexts where a particular outcome is certain, heuristics can aid decision-making. However, in novel contexts or when outcomes become otherwise uncertain or ambiguous, such heuristics could fail, requiring reanalysis. To prevent this, in cases of uncertainty, people become less sensitive to prior top-down heuristics: They suppress the use of previously informative cues and expend cognitive effort to reduce uncertainty. In other words, when people encounter uncertainty, they should lower the anticipation of an expected reward. Task performance may become more variable as people try new strategies to learn more about the context and seek out further information that could be used to make inferences (Hsu et al., 2005; Yu & Dayan, 2005; see also, Kosciessa et al., 2021).

Neurally, decision-making in the face of uncertainty is thought to involve a fronto-striatal network with differential involvement for unexpected and expected uncertainties (Elliott et al., 2003; Hsu et al., 2005; T. Wu et al., 2020). This network interacts with broader networks involved in cognitive control, including the frontal-parietal network and the cingulo-opercular network (including anterior cingulate cortex, supplementary motor area, and insula; T. Wu et al., 2020). Recent evidence suggests that the thalamaus may play a central role in cortical shifts that occur during decision-making under uncertainty (Kosciessa et al., 2021). To give one example, when comparing situations with unexpected uncertainties, where there is risk that is unknown beforehand (e.g., a deck of cards where probabilities are unknown; also called *ambiguous choices*), to situations with expected uncertainties, where risk is known beforehand (e.g., a familiar deck of cards where probabilities are known; also called *risky choices*), there is differential activation of frontal (orbitofrontal cortex) vs. striatal (basal ganglia, caudate) areas. Expected uncertainties appear to activate striatal systems, whereas unexpected uncertainties down-regulate the striatal system and up-regulate orbitofrontal cortex (Hsu et al., 2005). The two types of uncertainty also involve different neurotransmitters that are thought to optimize learning and decision-making, with unexpected uncertainties regulated by norepinephrine and expected uncertainties regulated by acetylcholine (Yu & Dayan, 2005). Correspondingly, expected uncertainties are thought to rely on model-based, top-down mechanisms whereas unexpected uncertainties are thought to down-regulate model-based mechanisms in favor of bottom-up mechanisms.

### Uncertainty in Language

In the traditionally separate domain of language, the notion of uncertainty has also been a central concept by way of ambiguity. Ambiguities can occur within a language at many levels of linguistic representation. For example, we encounter ambiguous words with multiple meanings, such as the word *bank* in English, which could refer to the edge of land near a body of water or a financial institution. Ambiguities can occur at other levels of processing as well, such as in phrasal attachment at the syntactic level. In the sentence *The man threatened the student with the knife*, the prepositional phrase (*with the knife*) can either attach to the first noun phrase (*the man*) or the second noun phrase (*the student*) leading to interpretations where either the man or the student is carrying the knife.

For many readers, these types of ambiguities go unnoticed, because they tend to have a preferred or expected reading. Occasionally expected readings go awry, resulting in amusing interpretations of sentences or news headlines. In the case of the headline *woman pushes brown bear as it climbs over fence to save her dogs*, many readers may have been left wondering what the woman did to her dog that prompted a bear to intervene. A key focus in psycholinguistics has been to investigate how people resolve these types of ambiguities and misinterpretations in the moment during comprehension and production. Do comprehenders simply rely on a strict set of processing heuristics to reduce memory burden and interpret a sentence (Frazier, 1979; Gibson, 1998), or do they use all available information in the context to make a flexible parse (MacDonald & Seidenberg, 2006; Trueswell et al., 1994)? Generally, there is evidence for the use of both heuristics and contextual integration, which can be captured by information theoretic perspectives centered on the tracking and updating of uncertainty (Levy, 2008; Levy et al., 2009). Here, bilingualism provides a unique perspective on this debate because languages tend to differ in their attachment preferences, and thus readers experience competition between their languages in terms of the best parse. There is evidence that exposure and the behavioral context matter, with observations that the parsing heuristics in an individual’s native language can shift toward their preferences in their second language after a period of immersion (Dussias & Sagarra, 2007). While it may be tempting to consider bilingualism as a special case of language processing, this would be unwise because it is estimated that over half the world’s population knows more than one language. Thus, in order to develop a more complete understanding of language and cognition, we should consider the full diversity of individuals, from monolingual to bilingual. Uncertainty is one approach that could capture this range of diversity in a general manner.

### Uncertainty for Bilinguals

People who are bilingual must cope with all the uncertainties and ambiguities raised above that occur within a language. Crucially, they experience an additional set of language-related uncertainties as well, namely those that occur across languages. Again, these ambiguities occur at various levels of linguistic representation, including the lexical (e.g., Duyck et al., 2007; Gullifer et al., 2013; Libben & Titone, 2009; Van Hell & Dijkstra, 2002) and syntactic (e.g., Bernolet et al., 2007; Dussias & Sagarra, 2007; Loebell & Bock, 2003) levels, but are most frequently studied at the lexical level. For bilinguals, nearly every concept can minimally be ascribed to a word in each language, and word forms can be ambiguous across languages. For example, in Spanish, *un vaso* is a drinking glass, but the word form looks strikingly like the English word *vase*. While these concepts are distinct, even highly proficient bilinguals experience momentary competition between conflicting meanings in the irrelevant language during spoken comprehension (Titone et al., 2020; Van Hell & Dijkstra, 2002), written comprehension (Gullifer et al., 2013; Gullifer & Titone, 2019), and production (Dussias et al., 2016; Gullifer et al., 2013). Managing this competition depends on individual differences in language exposure and cognitive control abilities (Gullifer & Titone, 2019; Kroll et al., 2013, 2015, 2016; Pivneva et al., 2014).

Competition between languages is similarly evident when bilinguals are tasked with switching between their languages (e.g., Meuter & Allport, 1999). A frequent observation from forced language switching tasks is that trials requiring a switch in language are associated with a processing cost relative to nonswitch trials. Often, but not always, these switches are asymmetric in nature, where it is more difficult to switch to the, often dominant, native language and easier to switch into the less dominant second language. This counterintuitive finding is taken as evidence that bilinguals apply a form of control (e.g., inhibition) to the unintended language which must be overcome when switching into that new language. Because suppression of the dominant language requires stronger inhibition than that of the less dominant language, it is harder to switch back to that language after it is suppressed.

At the same time, language switching costs can be linked to language-related uncertainty. In fact, one of the earliest papers on language switching characterized costs as arising from stimulus and response uncertainty (Macnamara et al., 1968). Importantly, language switching tasks are not commonly reflective of how language is actually used. Instead, they typically investigate lexical processing (production or comprehension) in a decontextualized manner, where switching occurs between isolated words and where the probability of switching is artificially controlled by the experimenter. Thus, the average language switching task could be considered a highly uncertain situation for participants, albeit one where the probability of switching becomes known over the course of the task. In contrast, naturalistic language switching, as occurs in bilingual communities, tends to follow observable patterns established by community language practices, which may function to reduce uncertainty.

In line with this view, psycholinguistic studies find that switching costs can be modulated by a variety of situations (see Bobb & Wodniecka, 2013, for a review). For example, unbalanced bilinguals are more likely than balanced bilinguals to exhibit asymmetric costs between languages (Costa & Santesteban, 2004; Meuter & Allport, 1999). These bilinguals may, on average, participate in “low entropy” language environments, where the less dominant language is relatively unlikely and benefits from strong suppression of the dominant language. In contrast, balanced bilinguals may have adapted to higher entropy language situations in which both languages are likely. Asymmetries or costs are also attenuated when more time is allotted to process the switch (e.g., Verhoef et al., 2009), when bilinguals are allowed to switch at their own will (e.g., Gollan & Ferreira, 2009), when switches are placed in sentence context (Gullifer et al., 2013; Ibáñez et al., 2010), and when language switches follow linguistic patterns that conform to the patterns of switching in a community (e.g., Beatty-Martínez & Dussias, 2017; Guzzardo Tamargo et al., 2016). All of these situations might be characterized as reductions in language-related uncertainty, and some may more closely approximate naturalistic language switching situations.

Still, in naturalistic environments, bilinguals are compelled to make decisions about which language or languages will come next, and they constantly face a set of questions linked to language-related uncertainty. Which of my languages do I speak with whom in the moment? Should I choose a language I am less comfortable in to accommodate my conversational partner, or would I express myself better with my most comfortable language at the risk of my partner failing to understand? Will I be judged for my choice of language (politically, academically, intellectually)? In some cases, the answer to these questions is that both languages are acceptable, and people will flexibly engage the entirety of their linguistic repertoires, as in the case of code-switching (Lipski, 1977; Poplack, 1980) or translanguaging (García & Wei, 2012; Williams, 1994).

Language-related uncertainties start early and can be pervasive throughout the lifespan. Even young children are aware of the social consequences of choosing a particular language or dialect, as when Lambert (1967) recounts his multilingual daughter’s hesitancy to invite two friends who speak different dialects for a ride to school. His daughter fears that inviting both friends would force her to show a linguistic preference for one friend or the other. In some cases, bilingual children as young as eight years of age may be called on to broker for their parents in high-pressure situations, where they must translate complex information beyond their years (e.g., legal or medical contexts). Brokering can have long-lasting cognitive and emotional consequences (López, 2020; López et al., 2020). Thus, bilinguals routinely encounter language-related uncertainties that depend on several factors, including the interlocutors, the communicative context, and individual preferences and proficiencies.

To begin to measure language-related uncertainties at a global level, we have developed a methodological approach based on information theory (Gullifer et al., 2018; Gullifer, Kousaie, et al., 2021; Gullifer & Titone, 2020a). Specifically, we use *language entropy* as a means to estimate language diversity and language-related uncertainty using questionnaire data. Similar entropy measures have also been used to quantify language diversity among multilingual twitter users (Eleta & Golbeck, 2014), within text-based code-switching corpora (Guzmán et al., 2017), and for diversity in choice of programming language use among software developers (Krein et al., 2009). We have shown that language entropy varies across communicative contexts within the same speakers and relates to differences in executive control engagement and language proficiency (Gullifer, Kousaie, et al., 2021; Gullifer & Titone, 2020a, 2020b).

## ADVANTAGES OF THE UNCERTAINTY APPROACH TO BILINGUALISM

In our view, a focus on uncertainty has the potential to mutually benefit and more closely integrate multiple subdomains of cognitive science, including decision-making, language science, and bilingualism. Attention and decision-making literature emphasize the role of uncertainty in behavioral contexts, and bilingualism can provide researchers with new ways of assessing contextual uncertainties through language. *Behavioral context* is defined as “a set of stable statistical regularities that relate the myriad environmental entities, such as objects and events, to each other and to our sensory and motor systems” (Yu & Dayan, 2005, p. 681). Thus, the uncertainty within a context can be quantified as a function of these complex features and interactions. Typically, contexts involve the entities and parameters within an experimental task, such as probabilistic cueing tasks, attention shifting tasks, betting-style card games, and generalizations of these tasks (e.g., Feldman & Friston, 2010; Hsu et al., 2005). These tasks often contain cue-target relationships (or other probabilities) that are known or learned over the course of the task and can be perturbed (or made ambiguous) in various ways, allowing for the investigation of risk and ambiguity. Crucially, the concept of behavioral context has been extended beyond isolated tasks into social psychological contexts (FeldmanHall et al., 2015, 2018; FeldmanHall & Shenhav, 2019), and it may apply in a broader sense to the social environments that people engage in during their daily lives in their communities. Thus, out in the world, uncertainties exist, fluctuate, and interact across many levels, from personal to ecological to societal (see the Systems Framework of Bilingualism, developed in Tiv et al., 2021 and the topic of a keynote invited by *Bilingualism: Language and Cognition* [Titone & Tiv, 2021]). Ultimately, one of the goals of cognitive science is to explain and make predictions about these types of naturalistic phenomena.

The notion of behavioral context is central to many usage-based theories about language and bilingualism, because people perceive and produce the various languages that they know with interlocutors in their environments (such as at home or in the workplace). This rich contextualization of language has wide-ranging consequences for language fluency, processing, representation, and control, and it may also carry consequences for domain general cognitive control and underlying brain mechanisms (Adler et al., 2020; Anderson et al., 2018; Beatty-Martinez et al., 2020; Green & Abutalebi, 2013; Grosjean, 2001, 2016; Gullifer & Titone, 2020a, 2020b; Hofweber et al., 2020; Tiv et al., 2020b). To give one example, the adaptive control hypothesis (Green & Abutalebi, 2013) posits that language usage within particular *interactional contexts* will have adaptive consequences for control and brain organization, where interactional contexts consist of the “recurrent pattern of conversational exchanges within a community of speakers” (Green & Abutalebi, 2013, p. 516). This notion is highly compatible with that of behavioral context from the cognitive literature. Green and Abutalebi delineate three specific types of contexts that are predicted to impact control processes recruited by language: single language contexts (where primarily one language is used), dual language contexts (where two languages are used and language switching occurs primarily between individuals), and dense code-switching contexts (where two languages are used and language switching occurs within individuals and within utterances).

Societies and communities may differ in aggregate along the lines of interactional context in ways that impact language and cognitive control. For example, Beatty-Martinez and colleagues have shown that otherwise comparable populations of highly proficient Spanish-English bilinguals differ in how they engage their languages. Participants living in Southern Spain tend to engage in single language contexts, while participants in Puerto Rico and mainland USA tend to exhibit behaviors associated with dual language or dense code-switching contexts (Beatty-Martinez et al., 2020). They further showed that these contextual differences had consequences for participants’ recruitment of cognitive resources for the purposes of language control.

We posit that contexts such as these differ with respect to language-related uncertainty, with dual language and dense code-switching contexts having higher uncertainty relative to single language contexts. The level of language-related uncertainty can be estimated, at a basic level, using entropy measures (Eleta & Golbeck, 2014; Gullifer & Titone, 2020a; Guzmán et al., 2017), either at the aggregate level (for a sample of participants), or as an individual difference measure (Gullifer, Kousaie, et al., 2021; Gullifer & Titone, 2020a; Guzmán et al., 2017). An even richer characterization can be provided by network scientific approaches (Eleta & Golbeck, 2014; Tiv et al., 2020b). Here, the entities in an environment and their interrelationships are modeled as networks using graph theory, allowing for a set of measures, including language entropy, to be extracted that provide information about the fundamental structure of an interactional (or behavioral) context.

Thus, researchers interested in uncertainty from a cognitive, attention, or decision-making perspective can exploit background language characteristics of participants as a sort of natural experiment. For example, the long-term role of behavioral context in cognitive adaptation can be investigated, between participants, by recruiting and contrasting participants who systematically vary in their language background in terms of interactional context (Beatty-Martinez et al., 2020; Gullifer et al., 2018; Gullifer & Titone, 2020b; Hofweber et al., 2020), providing a sort of naturalistic experiment. Within-participant comparisons can be made through longitudinal studies, for example, by recruiting samples of participants beginning their studies in a new (linguistic) environment and again several months later. Shorter term influences of behavioral context can be investigated by manipulating the interactional context of the experimental environment or interspersing cognitive tasks and language tasks that differ in language-related uncertainty (Adler et al., 2020; Hofweber et al., 2020; Y. J. Wu & Thierry, 2013). In sum, bilingual samples and their varied interactional contexts offer cognitive researchers a means to investigate adaptations that occur due to uncertainty in different behavioral contexts through observational and controlled experiments.

The neurocognitive study of uncertainty also has something to offer researchers interested in language and bilingualism. Namely, this perspective allows for an integration with computational, neurobiologically plausible models of cognition and control (Bastos et al., 2012; Friston, 2010; Yu & Dayan, 2005). For example, previously described entropy measures allow for a mathematical quantification of a range of uncertainties from language-related uncertainty with language entropy to uncertainty associated with task parameters. Uncertainty perspectives are inherently complementary to, and often explicitly couched in, Bayesian computational theories of cognition (Knill & Pouget, 2004). Such perspectives state that people maintain a set of prior beliefs about their behavioral contexts which figure into the decision-making processes. Priors are then adapted or optimized over time given exposure in the environment or behavioral context. Bayesian statistical models can be hierarchical, allowing them to capture the complexities of interactional contexts in a multilevel manner. Thus, with a Bayesian approach, prior language demands and uncertainties could be modeled simultaneously at the level of society, local communities, communicative contexts, and individuals. The tracking of uncertainty also has the benefit of being a neurobiologically plausible process (Feldman & Friston, 2010; Friston, 2010). For example, Friston (2010) applies an uncertainty perspective, the free-energy principle, to several brain theories, including the Bayesian brain hypothesis, efficient coding, and cell assembly theory.

In sum, by merging these perspectives within the general framework of uncertainty, we can more tightly contrast uncertainty at two levels: local, in the moment uncertainty and global uncertainty in the environment. Thus, the demands and processes involved in resolving local uncertainty must take into account the properties of the global or historical context. This is an essential link between general cognitive studies and linguistic approaches that examine how the sociolinguistic demands impact local psycholinguistic processes. Next, we provide an example of how uncertainty can be applied to the neurocognitive study of bilingualism by reviewing two “case studies” in this domain.

### Case Study #1: Language Entropy Captures Language-Related Uncertainty

We have used a measure of language entropy as a first approximation of language-related uncertainty that individuals encounter in their day-to-day environments, as a way to approximate interactional context. Language entropy is computed using Shannon entropy (Shannon, 1948), *H* = − $\sum_{i=1}^{n}$ *P*_*i*_log_2_(*P*_*i*_). Here, entropy (H) is computed over the proportion of usage for a particular language (*P*_*i*_) in a set of languages (*i* = 1 to *n*, where *n* reflects the number of languages in the set). The process can be repeated for any number of communicative contexts. Proportional usage is derived from self-report questionnaire data commonly collected in the field, such as language use in the home versus language use at work (Gullifer et al., 2018; Gullifer, Kousaie, et al. 2021; Gullifer & Titone, 2018, 2020a, 2020b). Importantly, the entropy measure is highly flexible and can be adapted to a range of data sources with a range of different language sets and states (including objective observations of language practices; e.g., Guzmán et al., 2017).

Language entropy can be thought of as providing a continuous index of language diversity or language-related uncertainty for a particular communicative context (or individual), with a range from 0 to some maximum value. Language entropy is at its minimum (*H* = 0) when one language in a set is used all the time in that context (i.e., 100% of the time) and the other languages never occur. A person with minimum language entropy in a context can be quite certain that a particular language will be used, and they should experience low levels of language-related uncertainty in this situation. The occurrence of the predictable language would also carry little information, as it reflects business as usual. However, the spontaneous use of another language would be highly unusual and convey information of some form.

**Figure 1.**
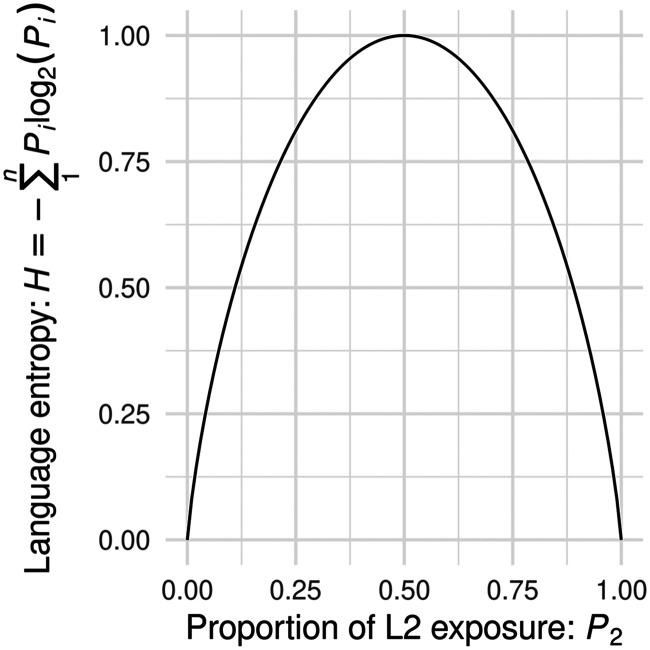
Relationship between L2 exposure (proportion) and language entropy for a hypothetical bilingual individual / communicative context. Language entropy is computed using Shannon entropy (Shannon, 1948), *H* = − $\sum_{i=1}^{n}$ *P*_*i*_log_2_(*P*_*i*_). In this plot, entropy (*H*) is computed over a range of proportions (0–1) for each of two languages (*P*_1_ and *P*_2_). Language entropy is at the minimum (*H* = 0) when either language is used 100% of the time and the other is used 0% of the time (left and right ends of the horizontal axis). Language entropy is at its maximum, equal to the logarithm (base 2) of the number of languages (here, two languages; *n* = 2) when the percentage of usage for two languages is equal within a communicative context (i.e., 50%–50% for a bilingual individual). Language entropy extends flexibly to situations with more than two languages.

Language entropy is at its maximum when the percentage of usage for two or more languages is equal within a communicative context (i.e., *H* = 1 for a 50%–50% for a bilingual individual; *H* = 1.585 for a 33%–33%–33% for a trilingual individual). A person with maximum language entropy in a particular communicative context should experience high levels of language-related uncertainty in this situation because either language is equipotent. Figure 1 illustrates possible language entropy values for a bilingual individual or context.

**Figure 2.**
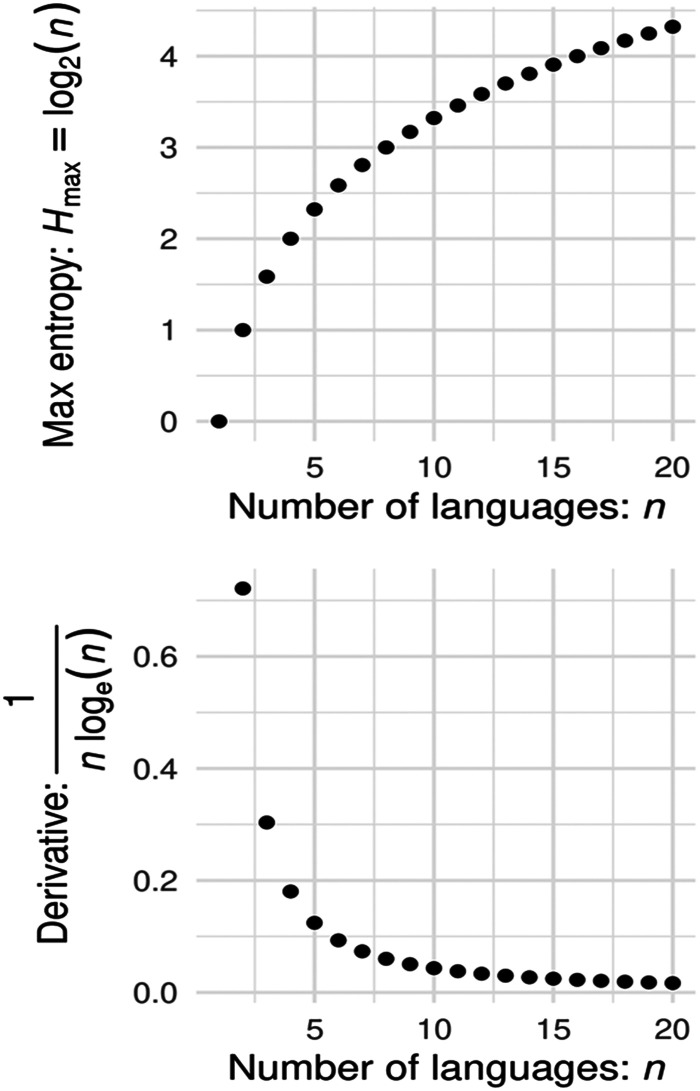
Mathematical relationship between possible maximum language entropy and the number of languages relevant for an individual or communicative context (top panel). Maximum entropy occurs when the proportion of usage is split evenly between the number of languages. Maximum entropy increases nonlinearly with the number of languages. The largest increase in possible maximum language entropy occurs when the number of languages shifts from one to two, observable in the top panel and illustrated in the bottom panel by the first derivative (rate of change with respect to the number of languages) of the language entropy function.

Mathematically, language entropy carries some interesting properties. The maximum possible language entropy for a context or individual increases as a function of the number of equally used languages (*H*_max_ = log_2_(*n*)), illustrated in Figure 2. Thus, the largest increase in maximum entropy occurs as the number of languages in a set increases from one to two (i.e., from monolingual to bilingual). This may reflect a boundary condition between monolingual language experience and bilingual/multilingual language experience. In other words, a monolingual individual who becomes bilingual has the possibility to experience a dramatic increase in language-related uncertainty. An equivalent increase would not be possible for a bilingual without the acquisition and usage of several additional languages. Moreover, while language entropy increases indefinitely as new languages are added to a set, there may be practical limits on language entropy that are imposed by a cap on the number of languages that highly multilingual people tend to use in their environments.

**Figure 3.**
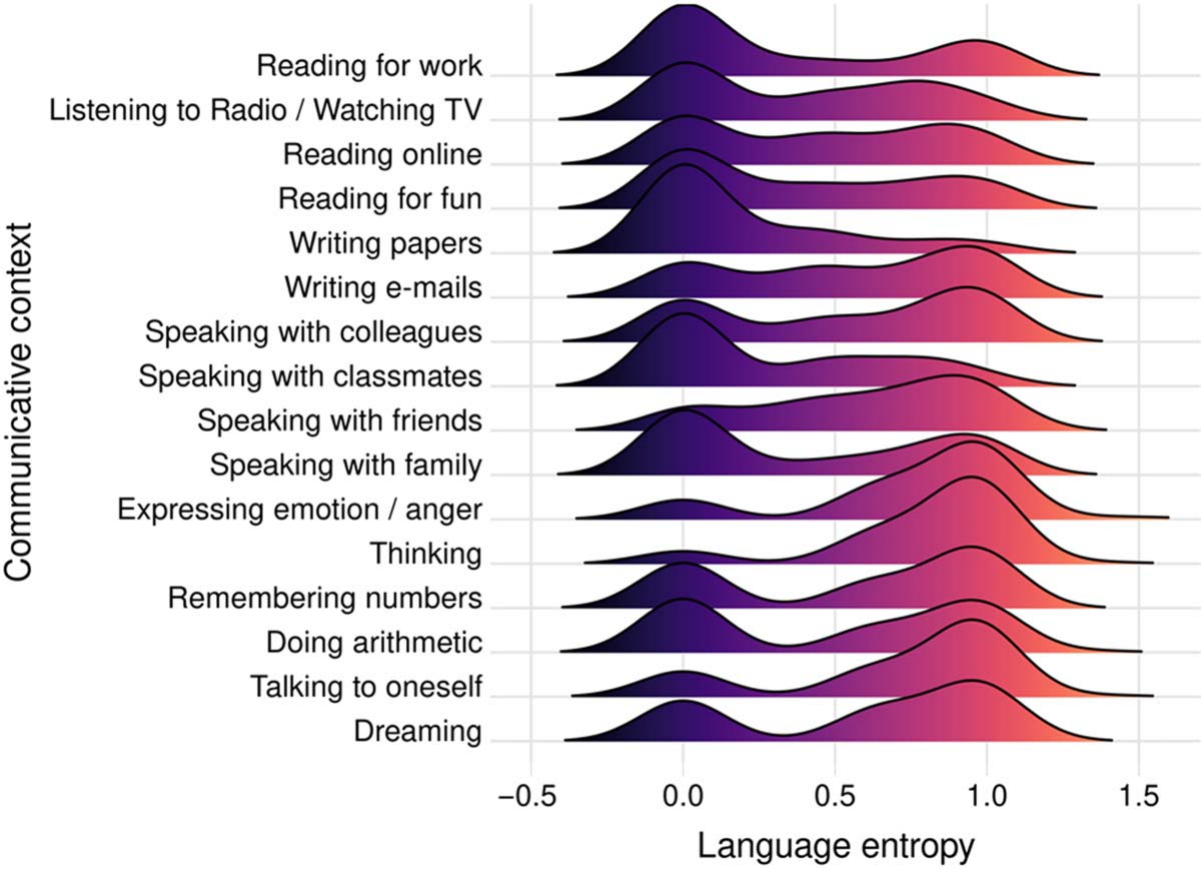
Illustration of the distribution of language entropy by communicative context. (Data adapted from Gullifer, Kousaie, et al., 2021.)

**Figure 4.**
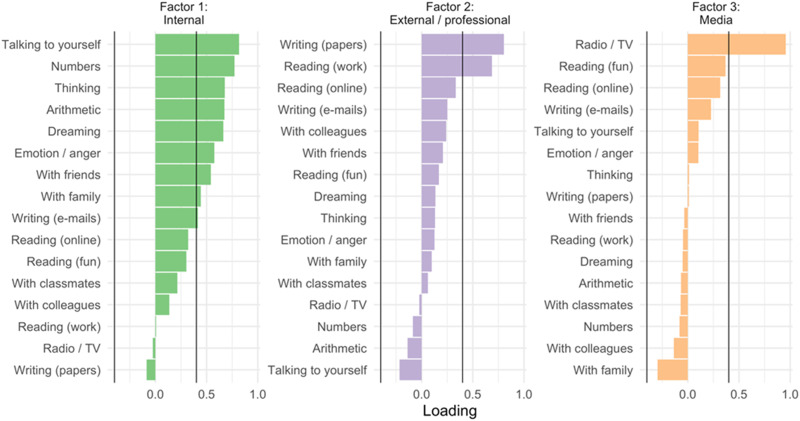
Illustration of the latent structure for language entropy. The vertical axis depicts each communicative context for which language entropy was computed. The horizontal axis depicts the factor loading. Each latent factor is displayed as a separate panel, encompassing language entropy for internal purposes, language entropy for external or professional purposes, and language entropy for media consumption. (Figure reproduced from Gullifer, Kousaie, et al., 2021.)

We have found that bilinguals and multilinguals living in Montréal exhibit individual differences in language entropy as a function of the communicative context (Gullifer, Kousaie, et al., 2021; Gullifer & Titone, 2020a), and that these contextual differences are captured by latent variable analyses. For example, Gullifer and colleagues (Gullifer, Kousaie, et al., 2021) probed language usage and language entropy across 16 different communicative contexts or domains (see Table 1 for descriptive statistics from that study and Figure 3 for an illustration of the distribution of data). Using factor analysis, they identified three latent domains of language entropy: entropy for internal aspects of language, entropy for external or professional aspects of language, and entropy for the consumption of media (see Figure 4, adapted from Gullifer, Kousaie, et al., 2021). Gullifer and Titone (2020a) observed a similar distinctiveness for language entropy in professional settings. More work is needed (with expanded language history questionnaires) to determine the ideal set of contexts within which to measure language entropy and to assess the consequences of moving between contexts. However, language entropy appears to provide a first approximation of the extent to which people jointly engage their two languages, on average, within their various communicative contexts. From an uncertainty standpoint, people with high language entropy, who report using two or more languages to an equal degree in their communicative contexts, likely experience higher degrees of language-related uncertainty in their daily lives that they learn to adapt to.

**Table 1.** Descriptive statistics for language entropy by language context from Gullifer, Kousaie, et al. (2021)

**Measure**	***M***	***SD***	**Min**	**Max**
Dreaming	0.60	0.42	0	1.15
Talking to oneself	0.71	0.38	0	1.39
Doing arithmetic	0.52	0.45	0	1.35
Remembering numbers	0.57	0.43	0	1.00
Thinking	0.80	0.30	0	1.39
Expressing emotion / anger	0.76	0.35	0	1.53
Speaking with family	0.41	0.42	0	1.00
Speaking with friends	0.61	0.35	0	1.13
Speaking with classmates	0.31	0.36	0	1.00
Speaking with colleagues	0.54	0.41	0	1.00
Writing e-mails	0.55	0.39	0	1.00
Writing papers	0.21	0.32	0	1.00
Reading for fun	0.39	0.40	0	1.00
Reading online	0.45	0.39	0	1.03
Listening to radio / watching TV	0.40	0.38	0	1.00
Reading for work	0.36	0.42	0	1.00

**Figure 5.**
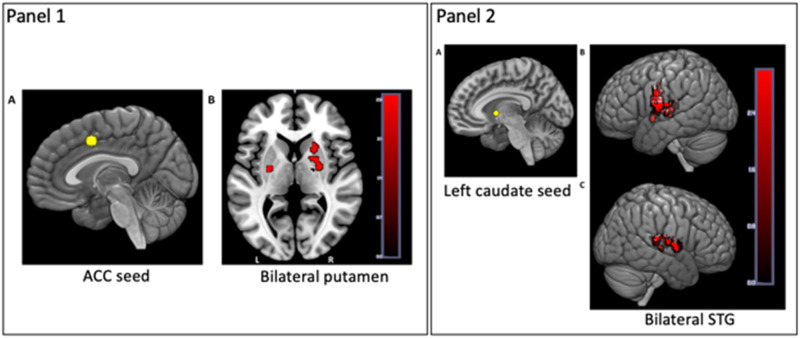
Depiction of the relationship between language entropy and resting-state functional connectivity. Language entropy (averaged across communicative contexts) is associated with greater resting-state functional connectivity between regions involved in language and control, particularly between ACC and putamen (Panel 1); and between left caudate and STG (Panel 2). ACC-putamen connectivity was, in turn, associated with greater reliance on proactive control in a behavioral task conducted outside the scanner. (Figure reproduced from Gullifer et al., 2018.)

**Figure 6.**
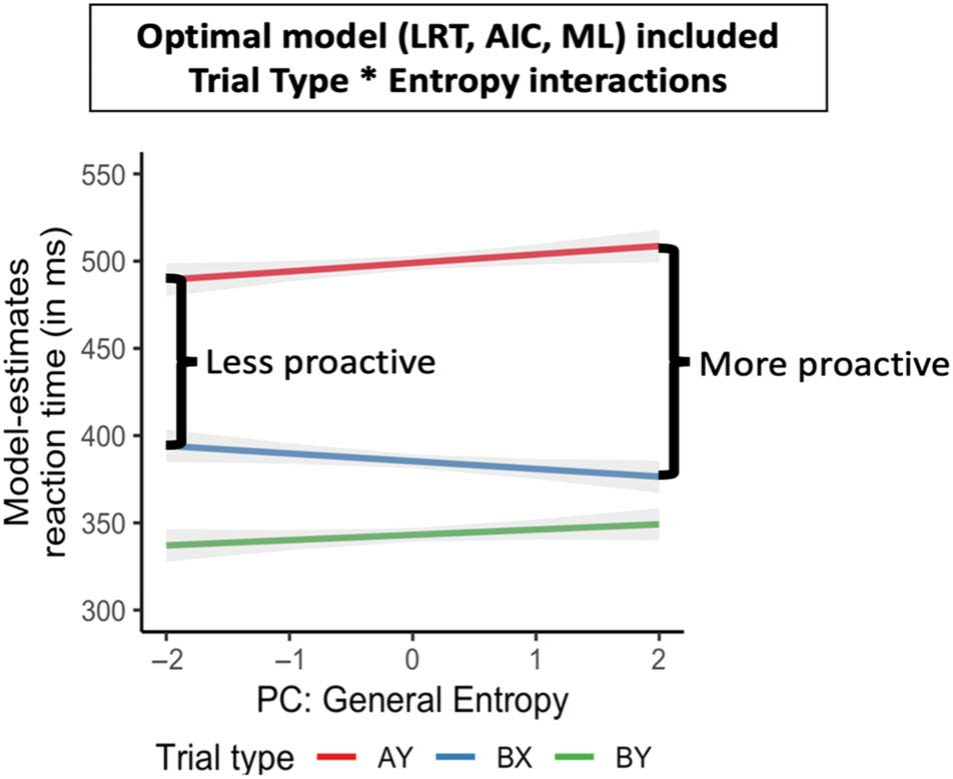
Depiction of the relationship between general language entropy and performance on the AX-CPT (reaction times). High general language entropy is associated with larger proactive cost scores (AY [red] vs. BX [blue]), signifying greater attention to goal-relevant information that is used in a proactive manner. (Figure reproduced from Gullifer & Titone, 2020b.)

Accordingly, we have found that individual differences in language entropy are related to neurocognitive aspects of executive control and language proficiency, suggesting that language-related uncertainty adapts the neurocognitive systems responsible for language and cognitive control. For example, individual differences in language entropy predict the functional organization of brain networks implicated in language and executive control (Gullifer et al., 2018) and aspects of language proficiency (Gullifer, Kousaie, et al., 2021; Gullifer & Titone, 2020a), as predicted by theories of neurocognitive adaptation and control (Abutalebi & Green, 2016; Green & Abutalebi, 2013). People with high language entropy (averaged over communicative contexts) exhibit greater resting-state functional connectivity among a network of areas associated with language and executive control (see Figure 5, adapted from Gullifer & Titone, 2018), and greater attention to goal-relevant cues that must be maintained to predict upcoming information in proactive control tasks like the AX-continuous performance task (AX-CPT; Gullifer et al., 2018; see Figure 6, adapted from Gullifer & Titone, 2020b). Comparable brain connectivity results have also been observed in another laboratory with a qualitatively different sample of bilinguals (Sulpizio et al., 2019), bolstering this method’s theoretical importance. Language entropy has been shown to relate to self-report and objective language proficiency (Gullifer, Kousaie, et al., 2021; Gullifer & Titone, 2020a), the ability to mentalize (or engage in social-cognitive processing) in the native and second languages (Tiv, O’Regan, & Titone, 2021), and other patterns of dual-language use such as engagement in language mixing (Kałamała et al., 2020).

On the one hand, the findings of proactive engagement of contextual information (and underlying brain networks) for high entropy bilinguals might go against the predictions of decision-making theories based on uncertainty, namely, that highly uncertain or ambiguous situations should down-regulate predictive mechanisms. However, these results can be explained under an adaptive mechanism in which participants who routinely experience high entropy environments may be better able to reduce internal uncertainty. We have speculated that bilinguals might adapt to contexts with language-related uncertainty by attending to other cues that are present in the environment. For example, phonetic or lexical cues encoded in the linguistic signal can preempt code switches; or particular interlocutors may have a tendency to use a particular language. These cues may be important for high entropy bilinguals who need to identify rapidly what language will come next to resolve language-related uncertainty in the environments at multiple levels.

There is also a possibility given our reading of the uncertainty literature, that high entropy bilinguals adapt to linguistically uncertain environments by creating a set of internal bilingual attractor states. For example, perhaps a new set of states is created that is related to a dual language (Green & Abutalebi, 2013) or a bilingual mode (Grosjean, 2001). Perhaps code-switching is a cognitive adaptation: an additional state that allows for the reduction of internal uncertainties for bilinguals in highly diverse language environments. These internal attractor states may provide bilinguals with an avenue for resolving language-related uncertainties during language processing in terms of generating predictions about what type of information will come next. If these possibilities are true, then language entropy (as a measure) may underestimate the diversity of language states, particularly for high entropy bilinguals. Other finer-grained methods may be able to more accurately estimate the diversity of language states. For instance, network science provides a means to measure entities and their interrelationships within an interactional or behavioral context.

### Case Study #2: Network Science Characterizes Behavioral/Interactional Context

While network models of multilingual language usage have been constructed from online sources, such as Twitter (e.g., Eleta & Golbeck, 2014), they have not, to our knowledge, been used to assess in-person, bilingual language usage. In a recent paper, we provide an example of how network science can be leveraged to uncover information about naturalistic language usage (Tiv et al., 2020b). We surveyed individuals about the languages that they use to discuss several topics of conversation (e.g., politics, sports, moral issues, religious issues) throughout different communicative contexts (e.g., at home, at work). We modeled these data as network graphs, in which topics of conversations were treated as nodes in a graph that were connected either by virtue of being discussed within the same context (and weighted based on the number of languages used to discuss these topics) or in the same language (and weighted based on the number of contexts they were discussed in). This allowed us to assess how topics of conversation co-occur within contexts and within languages.

**Figure 7.**
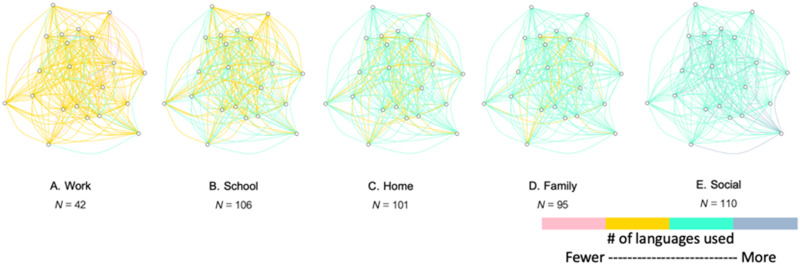
Depiction of the topic network for each of five communicative contexts. Nodes represent topics of conversation and edges indicate whether topics co-occurred in each domain. Edges are weighted by the total number of languages used to discuss two topics in a given domain: Green and blue hues indicate more languages, and pink and yellow hues indicate fewer languages. Topics that co-occur in work contexts tend to be discussed with fewer languages. Topics that co-occur in social contexts tend to be discussed with more languages. (Figure reproduced from Tiv et al., 2020b.)

In the context networks, we found that the various communicative contexts evidenced distinct configurations in terms of the topics that were discussed within those contexts (see Figure 7). In particular, few languages were used to discuss topics in the work environment, representative of highly compartmentalized language usage and low language-related uncertainty for these topics. In contrast, many languages were used to discuss the topics that occurred in individuals’ social contexts, representative of highly integrated language usage and high language-related uncertainty for these topics. In the language networks, we also found that there was greater specificity for the topics discussed in individuals’ less dominant language relative to the dominant language. Like the results for language entropy, the results here again confirm that language-related uncertainty can vary in a consistent manner according to the behavioral or communicative context.

We are now expanding the level of analysis to individuals’ language-tagged social networks (Tiv et al., 2020a) with the goal of assessing language usage for individuals (i.e., egos), between egos and their associates (i.e., ego-alter connections), and among their associates (alter-alter connections). Thus it will be possible to compute language entropy measures at these different levels (Eleta & Golbeck, 2014) and assess the extent to which they covary. Ultimately, we believe that the combination of language entropy and network science will be ideal for representing complex patterns of language practices and language-related uncertainty, as well as how these practices align with the language practices in their broader communities.

## SUMMARY AND NEW QUESTIONS

To sum up, we have brought together recent work showing how language-related uncertainty can be measured or estimated using language entropy and network science, and we have shown some of the interactions with other aspects of neurocognition, including language proficiency, brain organization, and proactive executive control abilities (Gullifer et al., 2018; Gullifer, Kousaie, et al., 2021; Gullifer & Titone, 2020a, 2020b; Kałamała et al., 2020; Sulpizio et al., 2019; Tiv et al., 2020b; Tiv, O’Regan, & Titone, 2021). This work is the beginning of a new paradigm in the domain of language science and bilingualism, and there are several aspects to be addressed going forward, related not only to measurement validity and generalization but also to linking theoretical domains and findings.

Measures like entropy and those computed from network analysis provide estimates of language-related uncertainty that are derived from self-report questionnaires. Future work should attempt to more closely approximate naturalistic language-related uncertainties through the use of objective measures such as corpus/dialogue analysis or the observation of naturalistic productions among bilinguals and multilinguals. Doing so will allow for further measurement validation and expansion of language-related uncertainty. For example, participants could complete language history or language-tagged social network questionnaires and then consent to having portions of their daily conversations recorded through a smartphone app. Or, they might respond to intermittent text message probes that inquire about language usage in the moment. Language entropy and usage patterns could be computed from the data elicited by these instruments. An advantage of a smartphone app or text message probes is that research could reach a broader and more diverse portion of the population than is typically sampled in experimental psychology.

Moreover, data from other geographic locations will be crucial in assessing the generalizability of these measures, methods, and theoretical perspectives. At the moment, only a few studies have assessed language entropy, most in the highly multilingual Montréal context (Gullifer et al., 2018; Gullifer, Kousaie, et al., 2021; Gullifer & Titone, 2020a, 2020b; Tiv, O’Regan, & Titone, 2021). However, there is emerging work from Italian (Sulpizio et al., 2019) and Polish (Kałamała et al., 2020) contexts as well. Thus, more research is needed before an initial sketch can be drawn across geographical locations and before we can determine the optimal level at which to measure uncertainty.

In terms of linking linguistic and cognitive perspectives (Feldman & Friston, 2010; Hirsh et al., 2012; Hsu et al., 2005; Peters et al., 2017; T. Wu et al., 2020; Yu & Dayan, 2005), going forward, we need to develop a greater understanding of how cumulative exposure to longer term environmental uncertainties interacts with shorter term local uncertainties in the moment, and how bilinguals represent and adjust to these uncertainties internally. This can be achieved by hierarchically integrating data at various levels from various sources, including macro social contextual information, such as language usage data present in population censuses; micro social contextual information, such as language usage data at the participant level; and local task-based information, such as language demands required by an experimental task in the moment. There are also links to be built with other domains that we only touched on briefly above, such as code-switching, learning, memory, and even mental health.

### Links to Code-Switching and Translanguaging

A crucial question is how bilingual practices such as code-switching or translanguaging fit with ideas of interactional context and language entropy. *Code-switching* is the practice of flexibly mixing languages (Lipski, 1977; Poplack, 1980). Sometimes languages are mixed between utterances, sentences, or interlocutors. Sometimes they are mixed within the same sentence (dense code-switching). The adaptive control hypothesis posits that dense code-switching contexts are theoretically distinct from dual language contexts, requiring the engagement of different control modes or cognitive mechanisms. However, in many ways dual language contexts could be viewed as a precondition for dense code-switching to occur. Code-switching tends to occur between bilinguals (who prefer to code switch) when the use of two languages is jointly viewed as acceptable, conditions that can be satisfied by a dual language context. While we have not assessed how language entropy relates to code-switching practices in Montréal, others have shown that rates of language mixing are higher for high entropy bilinguals (Kałamała et al., 2020), suggesting that the two are correlated. At the same time, not all bilinguals code-switch, even if they are continually exposed to highly integrated or uncertain (high entropy) linguistic environments. People who routinely engage in high entropy situations should develop internal attractor states that allow them to reduce internal entropy and predict upcoming information. For example, people could attract to a particular language state (e.g., either English or French) and default to a particular language; they could attract to a bilingual (French + English) state that results in frequent language switching between individuals or contexts; or they could attract to a code-switching state that involves frequent, dense code-switching. Here, there are likely be individual tendencies, but people may also be influenced by aspects of the social context, including their interlocutors (Kootstra et al., 2010).

*Translanguaging* is a perspective on bilingual language practices that is ostensibly similar to language switching (García & Wei, 2012; Williams, 1994). However, it characterizes language in a way that is distinct from typical conceptualizations in psycholinguistics, linguistics, and applied linguistics. These traditional perspectives tend to view languages as discrete entities in the environment. For example, although psycholinguistics shows evidence for cross-language activation during production and comprehension, and it often models the bilingual mind as massively integrated (e.g., Bernolet et al., 2007; Dijkstra & van Heuven, 1998, 2002; Hartsuiker & Pickering, 2008; Li & Farkas, 2002; Shook & Marian, 2012), there is a dominant focus on aspects like “native language” and “second language” and other individual traits, like proficiency, age of acquisition, and language dominance. These aspects are largely antithetical to translanguaging, which refers broadly to the language practices that bilinguals and multilinguals engage in. Translanguaging views languages as social constructs (largely imposed by monolingual majorities) as opposed to “ontologically real” entities (Makoni & Pennycook, 2007). Thus, in this perspective, language usage among bilinguals and multilinguals transcends the usage of individual languages, independently or jointly. In some ways, we view language entropy and (to some extent) network approaches as compatible with translanguaging. For example, entropy provides a measure that abstracts away from individual languages, and instead measures the diversity of or uncertainty associated with language usage. At the same time, in order to compute language entropy, information about usage of particular languages is elicited from participants, meaning that it is not completely abstracted away.

### Links to Learning and Memory

Mastering a second language is notoriously difficult, and recently the process of language acquisition has been characterized as a desirable difficulty (Bjork & Kroll, 2015; Kornell et al., 2009). A *desirable difficulty* is one in which there are initial costs to learning or performance that facilitate or enhance later learning. Desirable difficulties specifically engage the core processes involved in learning, comprehension, and memory. They include variable learning conditions (as opposed to predictable learning conditions), spaced study sessions (as opposed to mass study sessions), and interleaved practice (as opposed to blocked practice). Desirable difficulties have been applied to language learning through the observation that bilingualism often results in observable costs during language processing (thought to be the result of cross-language activation or competition) but other benefits in certain aspects of novel language learning (Kaushanskaya & Marian, 2009a, 2009b) and executive control abilities (Bialystok et al., 2012).

We note that several aspects of a desirable difficulty approach can be linked to notions of uncertainty. For example, inducing variable learning conditions and interleaving practice all function to increase uncertainty with respect to the nature of the task or learning environment. Moreover, in the uncertainty literature on decision-making there are suggestions that unexpected uncertainties in a new behavioral context encourage the exploration of new options, as participants try to identify the operative states that are conducive to task performance (Hirsh et al., 2012; Yu & Dayan, 2003, 2005). Thus, when faced with uncertainty, task performance becomes more variable and may encourage learning in the short term. Over the long term, learners may adapt their neurocognitive systems to expect or otherwise manage the types of persistent, ambient uncertainties that regularly occur in the environment (e.g., Beatty-Martinez et al., 2020). These adaptations could take many forms, including shifting expectations about altering linguistic material, altering cognitive control strategies, or incorporating code-switching or translanguaging practices. Ultimately these adaptations could allow for better control over language (Gullifer & Titone, 2020b) and changes in subjective and objective language proficiency (Gullifer, Kousaie, et al., 2021; Gullifer & Titone, 2020a).

However, there are issues to be resolved between an uncertainty perspective and a desirable difficulties perspective. For example, a key notion in desirable difficulties in language learning is that suppression of the native language plays a key role in the process of learning another (e.g., third) language (Bjork & Kroll, 2015; Bogulski et al., 2019). Thus, it may not solely be increases in general uncertainty that encourage language learning, but uncertainty that specifically involves the native language.

### Links to Language-Related Stress and Anxiety

Bilingual environments have been associated with language-related stress and anxiety for individuals who do not adapt to an immersion environment. This is shown primarily through social network analysis. The structural properties of individuals’ networks have implications for language proficiency, educational outcomes, and overall well-being. For example, when considering people who move to a new linguistic environment (e.g., students during study abroad or immigrants in a new country), social network structure (network size, density, interconnectedness) is positively associated with proficiency gains during language learning and educational outcomes (Baker-Smemoe et al., 2014; Doucerain et al., 2015; Gollan et al., 2015; Wiklund, 2002) as well as individuals’ overall sense of well-being. Notably, people with larger social networks during language immersion (i.e., networks from the host country) have fewer instances of language-related stress and depression (Church, 1982; Hendrickson et al., 2011). Inclusiveness and density of second language networks have been associated with the degree of communication-related stress in an immersion environment (Doucerain et al., 2015). In turn, a learner’s ability to cope with stressors is related to willingness to communicate and confidence in using that language: Students who are less burdened by stressors are more willing to communicate in a second language (Gallagher, 2013; MacIntyre et al., 2001). These results together suggest that a tight relationship between the properties of a learner’s social network, well-being, and willingness to use a language, and the proficiency gains made in that language. Thus, developing one’s social network expands opportunities for language use, and may force speakers to confront and adapt to various language-related uncertainties. Failure to adapt one’s internal representations to minimize uncertainty has been linked with stress, anxiety, and the occurrence of other diseases (FeldmanHall et al., 2015; Hirsh et al., 2012; Peters et al., 2017).

### Conclusion

Casting bilingualism as an exercise in managing language-related uncertainty has several benefits that can drive future research in various subdomains. As reviewed above, a focus on uncertainty allows for tighter integration between linguistic and computational cognitive theories that are neurally plausible. Such computational perspectives provide various metrics and measures that can be leveraged, including entropy. This integration will help in achieving common goals, such as investigating the impacts of behavioral context (global and local) on behaviors and brain organization. Ultimately, developing proficiency in a second language may be an exercise in reducing or adapting to uncertainty, allowing for efficient comprehension and production according to the behavioral or interactional context.

## FUNDING INFORMATION

Debra Titone, Natural Sciences and Engineering Research Council of Canada, individual Discovery Grant, Award ID: 264146. Jason W. Gullifer and Debra Titone, the Social Sciences and Humanities Research Council of Canada, Insight Development Grant, Award ID: 00935.

## AUTHOR CONTRIBUTIONS

**Jason W. Gullifer**: Conceptualization: Lead; Funding acquisition: Equal; Investigation: Lead; Project administration: Supporting; Writing – original draft: Lead; Writing – review & editing: Equal. **Debra Titone**: Conceptualization: Supporting; Funding acquisition: Equal; Project administration: Lead; Resources: Lead; Supervision: Lead; Writing – review & editing: Equal.

## TECHNICAL TERMS

Entropy: A concept from physics and information theory that quantifies the amount of uncertainty in a system, or the potential of a system to convey information.
Language entropy: An extension of entropy; it provides an estimate of language-related uncertainty for an individual or environment.
Behavioral context: The set of statistical regularities between objects and events within an environment; often refers to the properties of an experimental task.
Interactional context: The typical patterns of language use within a community of speakers; could be thought of as extending the idea of behavioral context to language.
